# Application of intelligent pacifying strategy information system in reducing short-duration MRI sedation rate in children

**DOI:** 10.1038/s41598-023-44049-y

**Published:** 2023-11-03

**Authors:** Xiaofang Deng, Deyi Zhuang, Jungang Liu, Cuimin Su, Xianghui Huang

**Affiliations:** 1 Children’s Hospital of Fudan University (Xiamen Branch), Xiamen Children’s Hospital, Xiamen, China; 2 Fujian Key Laboratory of Neonatal Diseases, Xiamen, China; 3 Jinjiang Municipal Hospital, Jinjiang, China

**Keywords:** Outcomes research, Paediatric research

## Abstract

Exploring and analyzing the effectiveness of an intelligent pacifying strategy information system based on assisted decision-making in reducing the sedation rate of children in short-duration magnetic resonance scans. A total of 125 children aged 3–5 years who underwent MRI scans at a children's hospital from July to December 2021 participated in this study, during which 62 children were assigned to a control group from July to September, and 63 children were assigned to an intervention group from October to December. In the intervention group, the pacifier used the intelligent pacifying strategy information system based on assisted decision-making to assess children's temperament, and utilization of a system-generated pacification plan according to assessment results. In the control group, the pacification plan was formulated by the pacifier based on their own experience and discussion with families of the participating children. The success rate of pacification, duration of pacification, and image quality of the two groups were compare. Compared with the control group, the intervention group had a higher success rate of pacification and lower duration of pacification, with statistically significant differences (*P* < 0.05). There was no difference in image quality between the two groups (*P* > 0.05). The intelligent pacifying strategy information system can help reduce the use of the sedative drugs in children aged 3–5 years who underwent a short-duration MRI scan.

## Introduction

Magnetic resonance imaging (MRI) is an imaging technique that uses magnetic fields and radiofrequency pulses to generate images of the human body^1^, which has the advantages of high soft-tissue contrast and no ionizing radiation. The use of sedative drugs can put the children in a sleep state temporarily so that they can complete the MRI scan, but children with immature physical development is prone to the occurrence of sedation-related adverse events, such as vomiting, agitation, hypoxia, and other complications^2,3^. To reduce the sedation rate in MRI scans, use of a pacification strategy before scanning to improve cooperation in non-sedated children is a research topic of interest within China and abroad in recent years^4^. Children aged 3–5 years whose cognitive development is limited and is in a phase of self-centeredness, have an attention span of 10–15 min^5^. Therefore, it is difficult for them to undergo a complex and long-duration MRI in a non-sedated state. However, their cognitive development allows for a certain understanding and ability for self-control. For ordinary canning sessions with duration of about 10 min in the conventional sequence short-duration MRI^6^, it is worth further studying whether the implementation of pacifying strategies can enable them to undergo MRI scans effectively without sedation. Assisted decision-making systems, which combine information systems and decision support technologies, can assist decision-makers in making evidence-based decisions, address the limitations in knowledge of medical staff effectively, as well as reduce decision bias and improve efficiency^7–9^. At present, most pacifying plans are formulated and implemented by pacifiers according to their experience. In this study, an intelligent pacifying strategy information system based on assisted decision-making was applied to reduce the use of sedative drugs in short-duration magnetic resonance scans in children aged 3–5 years.

## Method and materials

### Inclusion and exclusion criteria

A total of 125 inpatient and outpatient children aged 3–5 years who underwent magnetic resonance scanning in the radiology department of a children's hospital from July to December 2021 participated in this study, of which 65 were male and 60 were female. Participants were divided into a control group (62 cases) and an intervention group (63 cases) using a before-and-after controlled study method. Inclusion criteria: children aged 3–5 years; children who underwent brain, neck, and pelvis magnetic resonance imaging with a scan time of approximately 10 min (the sum of scanning time of each part and sequence in different directions^6^); informed consent from guardians of participants. Exclusion criteria: children with psychiatric disorders or epilepsy onset; children with intellectual disability.

### Pacifier responsibilities and qualifications

The pacifier was mainly responsible for the decisions in the control group and the implementation of the two groups, which is accompanying the children to play toys and games, and guiding the participating children to complete a simulated MRI scan and formal MRI scan^4,10^. The qualifications of the pacifier are: Bachelor’s degree or above; ≥ 3 years’ experience in working with MRI; successfully completing a hospital-based training program on play therapy; good verbal communication skills and organization and coordination skills in the pediatric population.

### Pacification process

**Figure 1:**
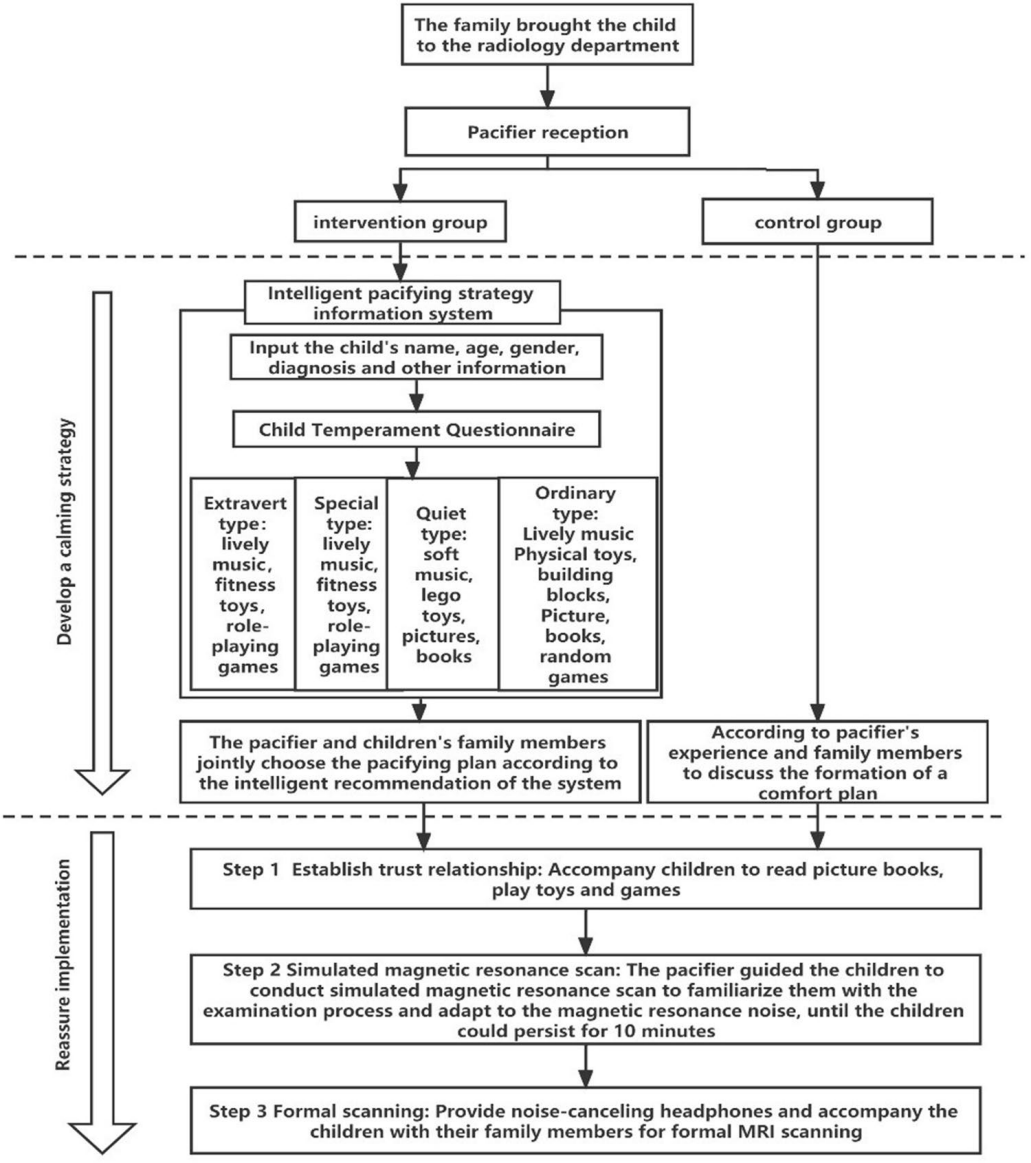
Pacification flow chart of the two groups of children undergoing MRI scan.

The appeasement process includes two steps: forming pacification programs and pacification implementation, as shown in the following parts and Fig. 1. Children who fail to pacify are sedated with chloral hydrate via enema, and the dosage of the medicine is 0.5 ml/kg.

### Forming pacification programs

In the control group, the pacifier drew up a pacifying plan which consisted of different choices with different aids such as music, toys, and games according to an assessment of the child’s family situation, personality characteristics, nationality, psychological state, educational level and other factors and from discussion with family members. In the intervention group, a self-developed intelligent pacifying strategy information system was applied by the pacifier which included temperament assessment and database. The intelligent pacifying strategy information system was established base on 306 cases collected in the early stage. And new data were collected for model validation and optimization. The temperament assessment included three steps: entering basic information, a temperament evaluation scale, and establishing pacifying plans.

The database contains a large number of children's temperament characteristics and various types of music, toys and games. Music is divided into two categories: light and lively, and soft and soothing. Toys include stamina, building blocks, clay and role-playing toys. Games include role-playing, peer interaction and building block games. Through artificial intelligence technology, the system can extract music, toys, and games suitable for children with different temperament types from the database according to the assessment results and generate a pacifying plan. After logging into the system, the pacifier enters the basic information of children such as name, age, gender, and diagnosis, followed by completing the temperament evaluation. In the temperament assessment step, the question of children's temperament assessment scale is answered by family members and filled out by pacifier. The pacifier asks the family members of the child questions according to the content of the temperament evaluation scale, and then checks the corresponding options of each question according to the answers provided. After completing the temperament evaluation, the system interface displays the evaluation result: the temperament type of the child. The system then extracts music, picture books, and games suitable for the child from the database and generates pacifying plans using artificial intelligence. Then the pacifier and the families discuss and decide which pacifying plan to take. The temperament assessment scale is the Parent Temperament Questionnaire (PTQ)^11^ using the questionnaire specific to children aged 3–7 years. There are 72 items in total, including nine domains: activity, regularity, avoidance, adaptability, reaction intensity, reaction threshold, emotional, attention span, and persistence. Its validity has been verified by many studies, and there was a study shows that the retest reliability of each dimension of the scale is: 0.48–0.77. The correlation coefficient of each dimension is − 0.29 to 0.54^12^. Children are divided into extroverted, special, quiet, and ordinary types, which determine their different preferences for toys and games^13^.

### Pacification implementation

#### Gaining children's trust and cooperation

1–2 family members were allowed to bring children for examination. Children and their families were greeted by the pacifier upon arrival at the radiology department and were accompanied to the pacification center, which is equipped with a picture book reading area and an interactive game area. With the children's favorite music as the background music, the children can read "Our Body", "The Amazing Brain" and other popular science books about diseases in the reading area, so that they can gain a better understanding of their bodies. The interactive game area provides children with a variety of toys and medical games, including slides, blocks, clay, and other toys, and including "accompanying" games, "know your body" games, "I am a little doctor" games. Through playing games, children can experience the process of medical diagnosis and treatment, so that they can understand the purpose and methods of the examination and be more cooperative with the MRI scan.

#### Simulated MRI scans

In the MRI simulation scan area, children watch a video of other children undergoing the MRI scanning process and then are guided through the MRI simulation scan. Firstly, the pacifier helps children lie down on the scanner bed and places a pair of noise-canceling headphone on them, which can reduce the noise to 50–100 dB, then plays the noise recording of MRI scan with a bluetooth speaker, which lasts for 10 min, and then accompanies children with their family for the scan, and reminds them to stay quiet and still. Through the simulated scan training, children can understand the structure of the MRI machine in advance, get familiar with the examination process, adapt to the sound of the MRI scan, and mitigate their level of fear. When children can stay still for at least 10 min during the simulated MRI scan, they will be taken into the MRI scanning room for the formal MRI scan.

#### Formal MRI scan

After the children enter the MRI scanning room, the pacifier put on the noise-reducing headphones for the children. Noise-reducing sponge material and a noise-reducing system are built into the noise-reducing headphones, which can reduce the noise of the MRI equipment by 20 dB during the examination, and provide a quiet MRI environment for children, and then accompanies children with their families for the formal MRI scan.

#### Evaluation indicators

The pacification success rate is defined as the number of cases of children completing formal MRI scans in a non-sedated state/total number of children in each group.

The duration of pacification refers to the time from the placation plan is determined until the formal MRI scan is completed. If pacification fails, the child is switched to sedative medication to complete the MRI scan, and the duration of pacification is not counted.

**Figure 2:**
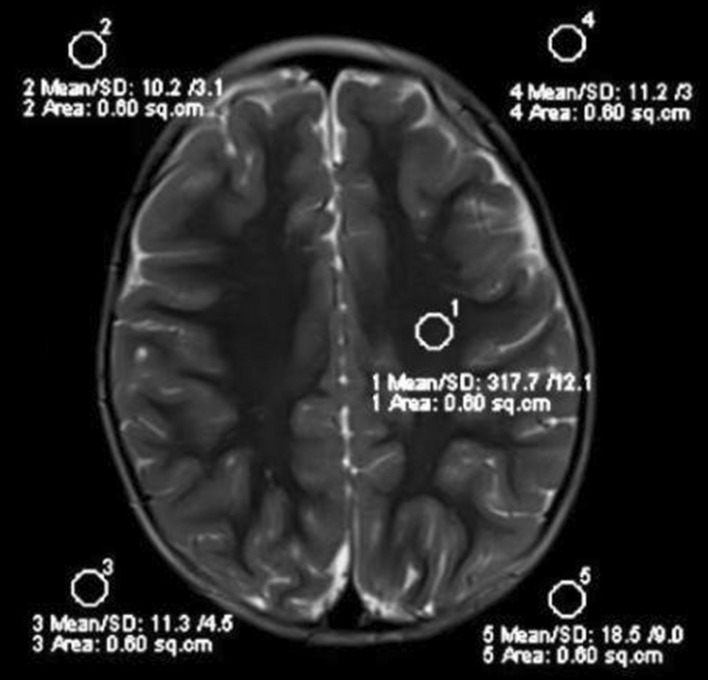
Measurement of ROI signal strength and standard deviation.

In this study, a Siemens 3T Skyra superconducting magnetic resonance scanner was used to collect images. The scanning sequence and scanning parameters of each inspection area are shown in Table 1. Image quality was compared using the objective evaluation index of signal-to-noise ratio^14^, signal intensity, and standard deviation of the region of interest (ROI) of clinical images at different sequences at each site, and the signal-to-noise ratio SNR = (S − Sb)/SD was calculated, with S denoting the mean ROI signal, Sb denoting the mean background signal, and SD denoting the standard deviation of the ROI signal, as shown in Fig. 2. The ROI signal-to-noise ratio was calculated, and the same method was used to draw the ROI of other locations at a uniform level, with their signal-to-noise ratios calculated separately, and finally the average of the signal-to-noise ratio was taken, followed by the signal-to-noise ratio of each image.

**Table 1:** Scanning site sequence and scanning parameters.

Parts	Sequence	TR (ms)	TE (ms)	FOV	Matrix	Depth (mm)
Head	T1WI	1800	9	230 × 230	320 × 320	5
	T2WI	4330	109	230 × 230	384 × 384	5
	FLAIR	8500	115	230 × 230	320 × 320	5
Neck	T1WI	690	9.1	180 × 180	256 × 256	2
	T2WI	2000	103	180 × 180	256 × 256	2
Pelvis	T1WI	700	10	380 × 380	448 × 448	5
	T2WI	4000	88	380 × 380	448 × 448	5

### Statistical analysis

EpiData 3.1 (EpiData Association, Odense, Denmark) was used to establish the database, perform double entries, and SPSS 20.0 (IBM Corporation, Armonk, NY) was used for statistical analysis. The measurement data were statistically described by mean ± standard deviation (x ± s), and the count data were expressed as a rate (%), and t-test and χ^2^ test were used to compare the two groups. Differences were considered statistically significant at *P* < 0.05.

### Ethical approval

This study was performed in accordance with the Declaration of Helsinki, and approved by the Scientific Ethics Committee of Children’s Hospital of Fudan University (Xiamen Branch), Xiamen Children’s Hospital ([2021] No.14). All guardian of participants signed the written informed consent to participate in the study.

## Results

A total of 125 children aged 3–5 years were included in this study. 65 males and 60 females underwent MR imaging of the head, neck in a flat scan mode, which took about 10 min. There was no significant difference in general data between the intervention group and the control group (*P* > 0.05), as shown in Table 2.

**Table 2:** Comparison of general data between the two groups.

Group	Cases	Age (months)	Sex		Scanning parts		
			Male	Female	Head	Neck	Pelvis
Intervention group	63	52.52 ± 9.69	32	31	35	10	18
Control group	62	53.15 ± 9.36	33	29	33	8	21
z/*χ*^2^		− 0.37	0.07		0.50		
*P*		0.72	0.79		0.78		

Compared with the control group, the intervention group had a higher success rate of pacification and lower duration of pacification, with statistically significant differences (*P* < 0.05), as shown in Table 3. A total of 10 children in the intervention group and a total of 16 children in the control group did not completed the MRI in the non-sedated state and needed to switch to sedation to complete the magnetic resonance examination.

**Table 3:** The success rate and duration of pacification compared between the two groups.

Group	The number of successful cases	Success rate (%)	Duration of pacification (min)
Intervention group	53	84.13 (53/63)	42.91 ± 5.43
Control group	46	74.19 (46/62)	54.35 ± 7.02
*χ*^2^/t		4.27	− 9.13
*P*		0.04	0.00

There was no statistical difference in image quality between the two groups (*P* > 0.05), as shown in Table 4.

**Table 4:** Comparison of image quality between the two groups.

Group	Cases	Head SNR (dB)			Neck SNR (dB)		Pelvis SNR (dB)	
		T1WI	T2WI	FLAIR	T1WI	T2WI	T1WI	T2WI
Intervention group	63	55.90 ± 3.48	61.61 ± 4.36	63.43 ± 3.41	67.88 ± 5.66	67.42 ± 2.35	63.53 ± 2.42	62.45 ± 2.58
Control group	62	55.92 ± 4.12	61.27 ± 4.16	62.87 ± 3.21	62.34 ± 5.80	65.18 ± 3.10	62.54 ± 2.20	61.32 ± 3.34
t		− 0.03	0.33	0.70	2.04	1.75	1.34	1.17
*P*		0.98	0.75	0.49	0.06	0.10	0.19	0.25

## Discussion

Studies have found that sedation can lead to various complications in children^2,3^. Children who complete MRI scans under non-sedation and reducing the use of sedation are areas of increasing interest in recent years. The use of sedative drugs during the MRI scan can be reduced by adopting pacification strategies^4,15,16^, such as providing a pacifying environment and with the use of a child’s favorite music, picture books, medical games, noise-canceling headphones, and the company of a pacifier and repeated MRI scans. Children aged 3–5 years have relatively limited cognitive development and are at a self-centered stage, unable to concentrate for long time, thus it is difficult for them to tolerate long-duration MRI scans without sedation. However, they have a certain comprehension and ability of control which make them capable of cooperation, it is more likely that they can complete short-duration MRI scans in the awake state. Therefore, whether the implementation of pacifying strategies during a short-duration magnetic resonance scan can reduce the use of sedative drugs effectively in this age group warrants further study.

Providing children with their favorite music, picture books, toys, games, etc. is conductive to effective communication and interaction between the pacifier and the children, reducing the child's fear and anxiety and improving cooperation and compliance at the same time. However, children with different temperaments have different behavioral, emotional, social, and interpersonal interactions, and have different preferences for music, toys, and games^12^. Children aged 3–5 years have relatively limited cognitive development and cannot express their emotions and needs well, and when decision-makers are faced with personal preference-sensitive decisions^17^, the results of the decisions are limited by personal experience, knowledge, and communication skills of the pacifier and parents. As a result, the selected pacifying measures such as music, picture books, toys, or games may not be appealing to the children, and the pacifier fails to obtain the trust and cooperation of the children, thus leading to an unsuccessful pacification process.

In the whole pacifying process, the pacifier plays an important role. There are studies through children's life experts who advise children before the examination and use age-appropriate techniques to pacify children during the examination. One study^18^ had established a professional pediatric team set up by radiologists, who will receive the patients and provide technical support for the children and pacify them. In this study, the pacifier not only provided professional counseling to the families before the examination, but also pacified the children with intelligent pacifying strategy information system. Information systems have been widely used in hospitals, among which auxiliary decision-making systems can improve the efficiency of decision-making and provide overall and multi-aspect support and knowledge services for decision-maker. A self-developed intelligent pacification strategy-assisted decision system was used in this study to evaluate the temperament type of children, and then the system analyzed and generated pacifying plans intelligently based on the assessment results for reference and selection by the pacifier and families of the children. Table 4 shows that there is no significant difference in the magnetic resonance image quality results of successful pacification between conventional pacification strategy and intelligent pacification strategy, but the results of Table 2 show that the number of successful children of intelligent pacification system is higher than that of the control group, and the pacification time is lower than that of the control group, indicating that the efficiency of intelligent pacification strategy is higher. The results of the study showed that compared with the control group, the intervention group had a higher success rate and required a shorter duration of reassurance, indicating that the assisted decision-making system can reduce the deficiencies of human experience and cognition, provide a more objective and comprehensive reference for the pacifier and families, improve the child's compliance and cooperation, thus reduce the use of sedative drugs during short-time MRI scans.

## Conclusion

The application of the pacification strategy system can help provide effective pacification plans and reduce the sedation rate of children aged 3–5 years who undergo short-duration MRI scans. In the future, multi-center research will be conducted to improve and upgrade the intelligent pacification system based on the psychological behavior characteristics of children and the knowledge base to better promote the application of pacification strategies in reducing the sedation rate of MRI scans.

## Data Availability

Data for the results of this study are available from the corresponding authors upon reasonable request.
